# A stepped-wedge cluster randomized trial designed to improve completion of HPV vaccine series and reduce missed opportunities to vaccinate in rural primary care practices

**DOI:** 10.1186/s13012-019-0871-9

**Published:** 2019-03-14

**Authors:** Patricia A. Carney, Brigit Hatch, Isabel Stock, Caitlin Dickinson, Melinda Davis, Rex Larsen, Steele Valenzuela, Miguel Marino, Paul M. Darden, Rose Gunn, Laura Ferrara, Lyle J. Fagnan

**Affiliations:** 1 Oregon Health & Science University, 3181 SW Sam Jackson Park Rd. MC: FM, Portland, OR 97239 USA; 2 Oregon Rural Practice-based Research Network, Oregon Health & Science University, Portland, OR USA; 3 Oregon Immunization Program, Portland, OR USA; 4 Department of Family Medicine, Oregon Health & Science University, Portland, OR USA; 5 OHSU & Portland State University School of Public Health, Portland, OR USA; 6 Oklahoma Child Health Research Network (OCHRN), University of Oklahoma Health Sciences Center, Oklahoma City, OK USA

**Keywords:** HPV vaccination, Cervical cancer prevention, Stepped-wedge cluster randomized trial, Practice-based research network, Cancer prevention social media campaigns

## Abstract

**Objectives:**

To test the effectiveness of a comprehensive team-based intervention to improve human papillomavirus (HPV) vaccination completion rates and reduce missed opportunities to vaccinate in rural Oregon.

**Design:**

Stepped-wedge cluster randomized trial.

**Participants:**

Forty family physicians and pediatricians who are members of the Oregon Rural Practice-based Research Network.

**Intervention:**

Tailored to individual practice needs, components will include (1) practice facilitation with clinicians, nurses, front office staff, and others who have patient contact to redesign patient care and communication strategies to optimize HPV vaccine series completion; (2) workflow mapping adapted to practice context to support HPV vaccine delivery; (3) a practice improvement model designed to firmly establish reminder and recall systems and then standing orders; (4) education for patients and parents that underscores HPV vaccination is safe, effective, and an important approach for reducing cancer risk; and (5) partnering with community organizations to plan and implement a social marketing campaign on HPV vaccination.

**Main outcome measures:**

Initiation and completion of the HPV vaccine series as well as reduction in rates of missed opportunities to vaccinate derived from Oregon Immunization Program data.

**Trial registration:**

ClinicalTrials.govPRS, NCT03604393: .Trial was registered on July 11, 2018. The first participant was enrolled on September 11, 2018.

## Precis

Given the low rates and wide range of HPV vaccine initiation and completion in rural settings, a critical need exists to support primary care practices and their communities toward improving HPV vaccination rates and identify successful strategies for future intervention.

## Background

The human papillomavirus (HPV) types in the current 9 valent vaccine account for about 73% to 88% of cancers of the cervix, vagina, and anus and for approximately 55–65% of cancers of the vulva, penis, and oropharynx [1, 2]. About $8 billion is spent annually to manage the sequelae of HPV infections, primarily to manage abnormal cervical cytology and cervical neoplasia [3]. This exceeds the economic burden of any other sexually transmitted infection except for the human immunodeficiency virus [3].

In 2006, the US Food and Drug Administration approved a vaccine to protect against HPV-16 and HPV-18 [4], which are most closely associated with cervical cancer. Though several versions of HPV vaccines have been available in the past, only Gardasil-9, which protects against strains of HPV most closely associated with cancers of the cervix, penis, vulva, anus, and oropharynx, is currently available in the USA. As HPV vaccination rates have increased, there has been a corresponding decrease in HPV infection and related cancers [5, 6]. Vaccination is currently recommended with a two-shot series for all children (males and females) ages 9–14 or as a 3-shot series for those 15 through 26 [7, 8]. The FDA also recently expanded licensure of Gardasil-9 (October 5th, 2018) to include adults up to age 45, though most professional bodies have not yet evaluated this option [9].

Unfortunately, the US National Immunization Survey (NIS) indicates many fail to complete the vaccine series [10]. Among females, national vaccine initiation among 13–17-year-olds increased from 53% in 2011 to 60% in 2014. However, national vaccine series completion was only 48.6% with rural completion 10% lower than urban areas and varyingly by state (28.8 to 78.0%) [10]. Among males, a modest increase in initiation occurred between 2014 and 2015 (from 41.7 to 49.8%), with completion rates increasing from 21.6 to 28.1% [11]. HPV vaccination coverage remains 36.5% lower than meningococcal ACWY (MenACWY) and 40.1% lower for tetanus, diphtheria, and acellular pertussis (Tdap) [10]. This disparity in vaccine uptake demonstrates a missed opportunity for HPV vaccination.

Many barriers have contributed to low HPV vaccination rates, including factors at the system (e.g., practice) and individual levels (e.g., provider, patient/family). One systematic review of 76 published studies [12] identified seven common barriers to practices, including lack of awareness, lack of familiarity with details, lack of agreement with guidelines, issues related to self-efficacy (ability to reach goals) [12], outcome expectancy (belief that given behaviors will lead to certain outcomes) [13], ability to overcome practice inertia, and external barriers such as health insurance coverage [14]. Patient and family-level barriers include decision to delay rather than refuse vaccination [15], concerns about vaccine safety, the perception that vaccination is unnecessary, and lack of a provider recommendation [16]. Importantly, patient, physician, and practice-level barriers are context-specific and highly variable [17], so improving HPV vaccination rates will require a tailored multi-component approach.

More rigorous research is needed to understand how to optimize HPV vaccination rates, especially in rural underserved communities. Toward this end, we developed a research proposal funded by the American Cancer Society (RSG-18-022-01-CPPB) to test, using a stepped-wedge cluster randomized design, a multi-component intervention to improve HPV vaccine completion rates and reduce missed opportunities to vaccinate. Table 1 illustrates summary information on trials conducted to date and what our specific intervention components will include. The overarching goal of the study is to engage rural primary care practices and community organizations to test interventions designed to increase HPV vaccination including initiation and completions in males and females aged 11–17 years, with an emphasis on 11–12-year-olds. The purpose of this paper is to describe the study protocol in detail.

**Table 1:**
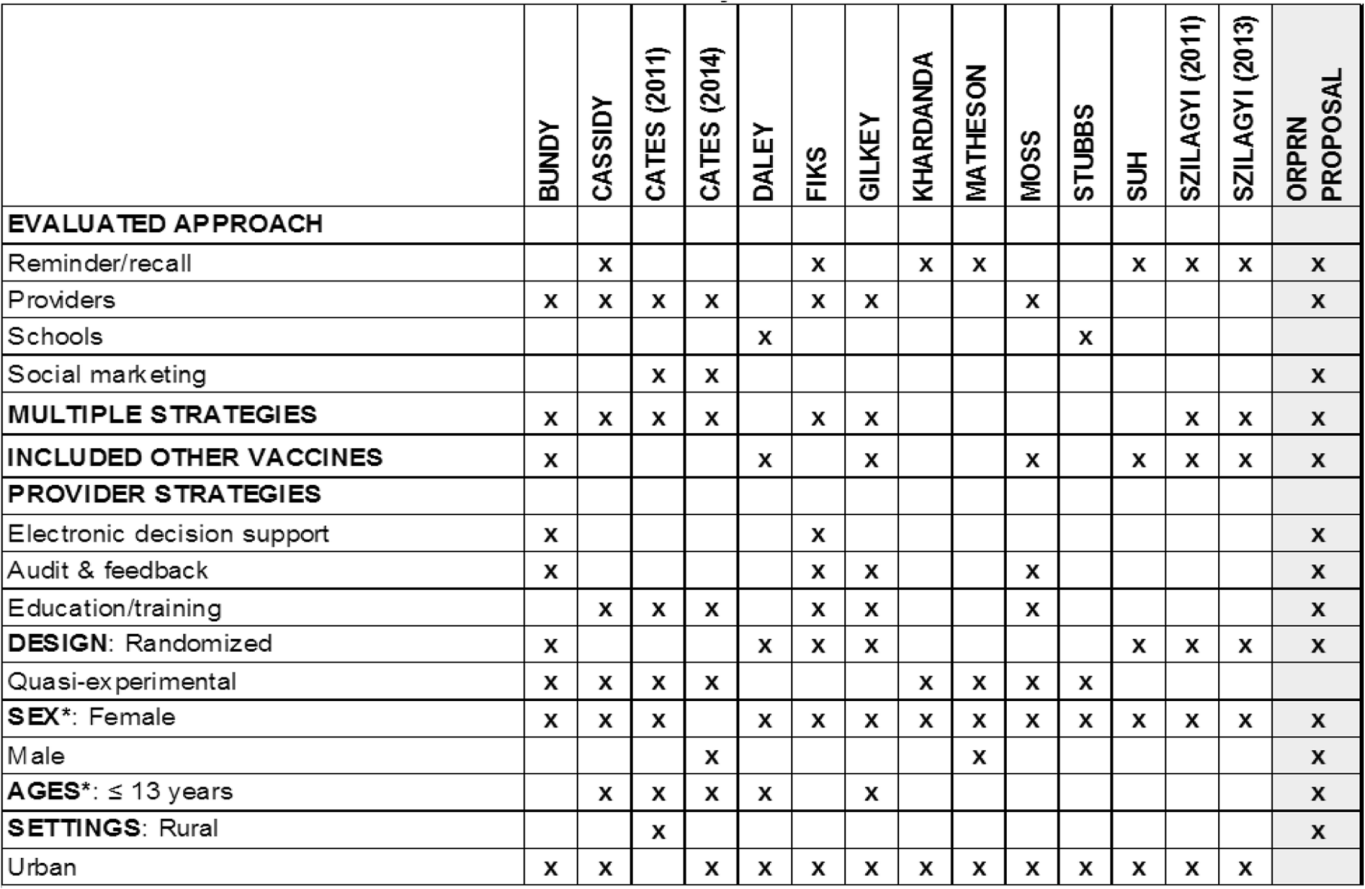
Characteristics of practice and community-based HPV interventions

Multiple strategies within or across approaches (overarching design): not necessarily evaluated separately

*Either restricted to specified group or reported findings separately for each group

## Methods

### Study setting and recruitment

Oregon Rural Practice-based Research Network (ORPRN) is based at Oregon Health & Science University (OHSU) with a mission of improving the health of rural Oregonians by promoting knowledge transfer between communities and clinicians [18]. ORPRN has built statewide relationships with primary care physicians, practices, hospitals, community groups, health systems, payers, and Accountable Care Organizations (ACOs). ORPRN reflects the geographic, demographic, and practice diversity of rural and underserved Oregon. ORPRN studies frequently link community-based public health with primary care practices, while also maintaining close relationships with the Oregon Health Authority (OHA), and health promotion and education organizations throughout the state, including the Oregon Immunization Program (OIP).

In this study, we will enroll eligible ORPRN primary care clinics located in rural communities (defined by Oregon Office of Rural Health as any geographic area in Oregon that is ten or more miles from a population center of > 40,000 people) that see adequate numbers of patients aged 11–17 years. Based on our sample size estimate, we will need 40 practices for the study. We will overenroll to 45 to account for an attrition rate of 11%. *We received over 60 letters of support from rural practices that expressed interest in participating.* Practice enrollment is currently underway. Community organizations established for at least 1 year and co-located in communities of participating primary care practices will also be eligible to take part. These will include Accountable Care Organizations (ACOs), which are healthcare organizations tasked with providing high-quality, low-cost care to their Medicaid and Medicare beneficiaries. In Oregon, the ACOs for the Medicaid population are 16 Coordinated Care Organizations (CCOs) [19].

### Study aims

**Fig. 1:**
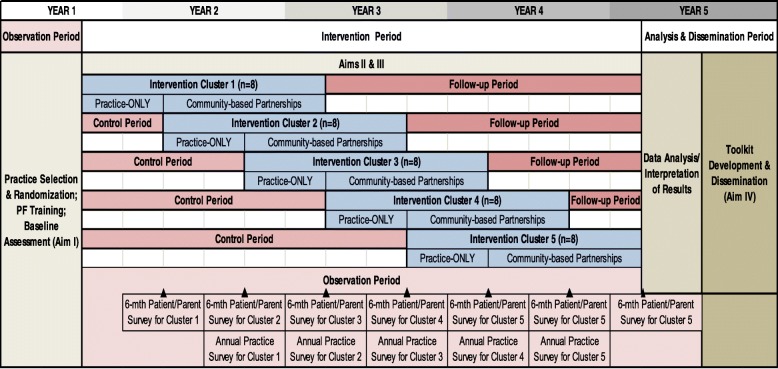
Study design

The study has four specific aims described below. Figure 1 outlines the study design and timeline.

### Overview of methods to address each specific aim

#### Aim 1: Conduct a baseline assessment of how primary care practices and community-based organizations are addressing initiation and completion of the HPV vaccine series

To address aim 1, we will use data on vaccine initiation and completion rates from OIP’s immunization registry, ALERT Immunization Information System (IIS), to identify 12–15 practices with both high and low HPV vaccine initiation and completion rates in rural Oregon family medicine and pediatric practices. After practices are enrolled, we will conduct direct observations using expert qualitative investigators (authors MD and RG) to assess practice systems and workflows associated with high and low vaccination rates. An iterative sampling framework will inform practice recruitment—we anticipate enrolling, using a 2:1 ratio, higher performing clinics relative to lower performing clinics. Additional study measures for aim 1 (Table 2) include a pre-site visit interview and a practice survey administered prior to the observation visit. Study staff will additionally assess public health programs and community-based organizations in the communities where the practices are located to assess community perceptions of the HPV vaccine and efforts in the community to address vaccination completion.

**Table 2:** Data collection methods, timing, and measures

Aim 1: Data collection methods, timing, and measures	
Pre-site visit phone interviews—once at baseline	Organizational structure, key stakeholders, practice champion
	Approaches to delivery of adolescent preventive care services (such as immunizations)
Formal site visit—once at baseline	Baseline workflows; baseline patient data collection: eligible patient population, patient demographics, and other characteristics of the patient population; baseline immunization rates; baseline community partnership data: existing education programs and efforts and existing partnerships; use of ALERT IIS and EHR to capture and track immunizations
Practice survey (PS)—once at baseline	Practice and practice’s patient demographics, practice change, payer mix, revenue and payments, and HPV vaccine priority
ALERT IIS—at baseline	Patient demographics (age, race/ethnicity, vaccination status, insurance status, and other covariates)
Aim 2: Data collection methods, timing, and measures	
Quality Improvement Change Questionnaire (QICA)—every 12 months	Engaged leadership
	Organized, evidence-based care; quality improvement strategy; continuous and team-based healing relationships; care coordination
Site visits—monthly, for 18 months	Workflows; patient data collection: eligible patient population, patient demographics, and other characteristics of the patient population; quarterly immunization rates; quarterly community partnership data: existing education programs and efforts; existing partnerships
Intervention data entries—monthly	PDSA cycle worksheets, workflow mapping, field notes
Practice survey (PS), administered as needed to reflect change	Practice and practice’s patient demographics, practice change, payer mix, revenue and payments, HPV vaccine priority
ALERT IIS—quarterly	Patient demographics (age, race/ethnicity, vaccination status, insurance status, and other covariates)
Aim 3: Data collection methods, timing, and measures	
Pre- and post-community partner group interviews	Acceptability of HPV vaccination
Patient/parent surveys—every 6 months	Child and parents’ knowledge about cancer risk reduction and the HPV vaccine series; where and how information about HPV and cancer risk reduction via vaccination was sought; demographic information

#### Aim 2: Implement and test, using a stepped-wedge cluster randomized trial design, the effectiveness of a multi-component primary care practice-based intervention on initiation and completion of the HPV vaccine series as well as reduction in rates of missed opportunities to vaccinate

**Fig. 2:**
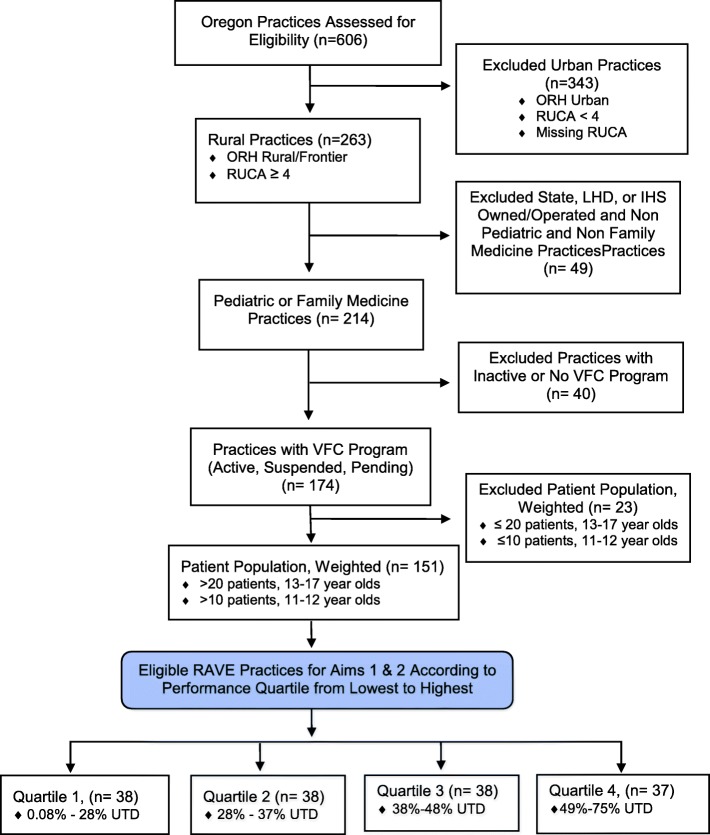
RAVE CONSORT diagram, practice eligibility—aims 1 and 2. *ORH* Oregon Office of Rural Health, *RUCA* Rural-Urban Commuting Area, *LHD* Local Health Department, *HIS* Indian Health Services, *VFC* Vaccines for Children program, *UTD* up to date on HPV immunizations

The stepped-wedge cluster randomized trial design affords many benefits, as statistical power can be gained by randomizing clusters of practices with correlated outcomes such as immunization rates to the intervention timing, while the remaining practices serve as waitlisted controls [20, 21]. Authors MM and SV will conduct block randomization of practices in five wedges (Fig. 1). Outcomes will be measured every three months in all practices at every period, so that each practice provides data points in both the control and intervention conditions. Prior to randomization, practices will be stratified based on designation of pediatric or family medicine clinic to balance pediatric/family medicine clinics at each wedge. To compare the effect of the intervention with usual care on HPV outcome measures in the context of a stepped-wedge design, we will utilize generalized linear mixed models (GLMMs) [22]. In addition to tracking enrolled study practices using data from the OIP, we will also track unenrolled comparable rural practices to describe secular trends and potentially serve as an additional comparison group to assess generalizability. The intervention will be conducted in 45 primary care practices (Fig. 1). Given this sample size, the stepped-wedge design with five cohorts of eights practices, two-sided significance level of 5%, starting practice-level HPV completion of 24%, intracluster correlation of patients within practices of 0.08, we will have at least 80% power to detect a 8.8% relative increase in HPV completion performance [23, 24]. Figure 2 illustrates our CONSORT diagram of included practices along with reasons for exclusion relevant for aims 1 and 2. Interventions are based on an adapted version of the Solberg Model, which posits that change is influenced by a complex interaction of factors both in and outside the practice, and interventions based on understanding these interrelationships can yield sustainable improvements in primary care practice [25]. Intervention components will be tailored to individual practice needs and will involve (1) practice facilitation with clinicians nurses, front office staff, and others who have patient contact to redesign patient care and communication strategies to optimize HPV vaccine series completion; (2) workflow mapping adapted to practice context to support HPV vaccine delivery; (3) a practice improvement model designed to firmly establish reminder and recall systems and then standing orders; and (4) education for patients and parents that underscores HPV vaccination is safe, effective, and an important approach for reducing cancer risk. We will additionally explore uptake of the intervention in terms of implementation timing and component features in practices with substantial and limited improvement to determine which practice characteristics affect delivery of the full HPV vaccine series. Study measures for specific aim 2 (Table 2) include a Quality Improvement Change Questionnaire (QICA), which will be administered routinely; a practice survey and staff member survey: quarterly ALERT IIS data regarding HPV vaccination rates (initiation and completion); monthly site visits conducted by study staff to assess study intervention process activities; and monthly intervention data entries from improvement cycles worksheets, workflow mapping, and field notes.

#### Aim 3: Explore the extent to which an evidence-based social marketing campaign, implemented in partnership with community organizations and participating practices, will increase HPV vaccination readiness across the community

In aim 3, the randomized primary care practices will select a community-level organization to partner with, such as a regional Accountable Care Organization (ACO) Community Advisory Council (CAC), patient and family advisory groups, or local public health programs. Together, the practice and community group will plan and implement an evidence-based community-level intervention [26, 27] designed to improve knowledge among adolescents and their parents about HPV, cancer risk, and risk reduction via vaccination, as well as the safety and effectiveness of the vaccine series. The plan specifically explores the extent to which this partnership brings “vaccine-ready” adolescent and parents to primary care practices and increases the demand for HPV vaccination [28]. Activities may include vaccine fairs, radio or newspaper ads, posters and brochures from Centers for Disease Control (CDC) website, resources for providers, such as websites, tips, and Frequently Asked Questions, and social media posts (Facebook, Twitter).

Study measures for aim 3 will include pre- and post-community partner group interviews on the acceptability of HPV vaccination and patient/parent surveys to assess child and parents’ knowledge about cancer risk reduction and the HPV vaccine series, where and how information about HPV and cancer risk reduction via vaccination was sought, as well as demographic information.

#### Aim 4: Explore the impact of sharing promising clinical and community intervention features in a toolkit with practices, state public health programs, and Accountable Care Organizations

To address aim 4, we will create a practice and community HPV vaccine improvement toolkit for use by practices and community partners. The toolkit will be disseminated throughout Oregon by ORPRN, OIP, and the American Cancer Society through publication and as an online resource. Determining the toolkit’s direct impact on HPV vaccination completion rates is beyond the scope of this study. However, OIP will track the number of electronic downloads of the toolkit and be able to link this to rates of HPV vaccine completion as an indirect assessment of improvements in vaccine completion rates due to the toolkit. Our national dissemination plan will include five key activities: (1) presenting the final toolkit and project at the National Immunization Conference, which brings together a variety of partners to explore science, policy, education, and planning issues related to immunization; (2) distributing the toolkit on the National VFC/AFIX Group listserv through OIP’s partnership. Subscribers to this listserv represent more than 260 state and local immunization program and immunization registry staff from the USA; (3) distributing the toolkit on the Oregon Immunization Partners listserv, which currently has 2900 public and private providers; (4) distributing the toolkit on the Oregon Public Clinic listserv, which includes more than 1100 public providers from health departments, federally qualified health centers, school-based health centers, and tribal practices; and (5) Putting the toolkit on the OIP website hosted by the state health department. The OIP website is a critical resource utilized by over 2000 providers, ACOs, Health Systems, payers, and members of the public.

### Preliminary data: findings from family medicine and pediatric practices in rural Oregon

With OIP, we identified specific HPV series initiation and completion rates from 2017 at the level of the county as well as the practices, and we have obtained county and practice-weighted population estimates for 13–17 year-olds (Table 3). We will utilize OIP’s previously validated practice weighting estimates [29] which reflect the likelihood that a patient is still present in a practice based on the length of time since the last vaccine was reported to ALERT IIS. In a preliminary analysis, we found that 47% of adolescents at the 53 primary care practices in rural Oregon that we included in this analysis have initiated the vaccine (practice-level range is 2–75%) and 24% of patients have completed the series (practice-level range 0–47%). These findings indicate that there is a need to develop and test interventions in these rural underserved areas to improve vaccination rates. We are confident in our multi-component intervention, and our study approach will rigorously test its effectiveness.

**Table 3:**
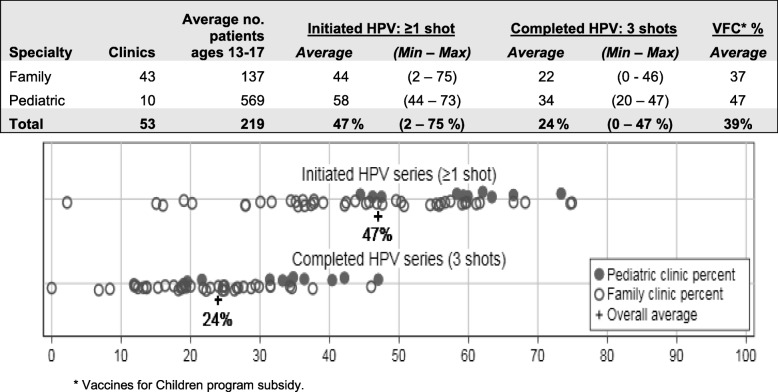
2017 HPV vaccination initiation and completion rates among rural Oregon practice (*n* = 53 practices)

*Vaccines for Children program subsidy

**Fig. 3:**
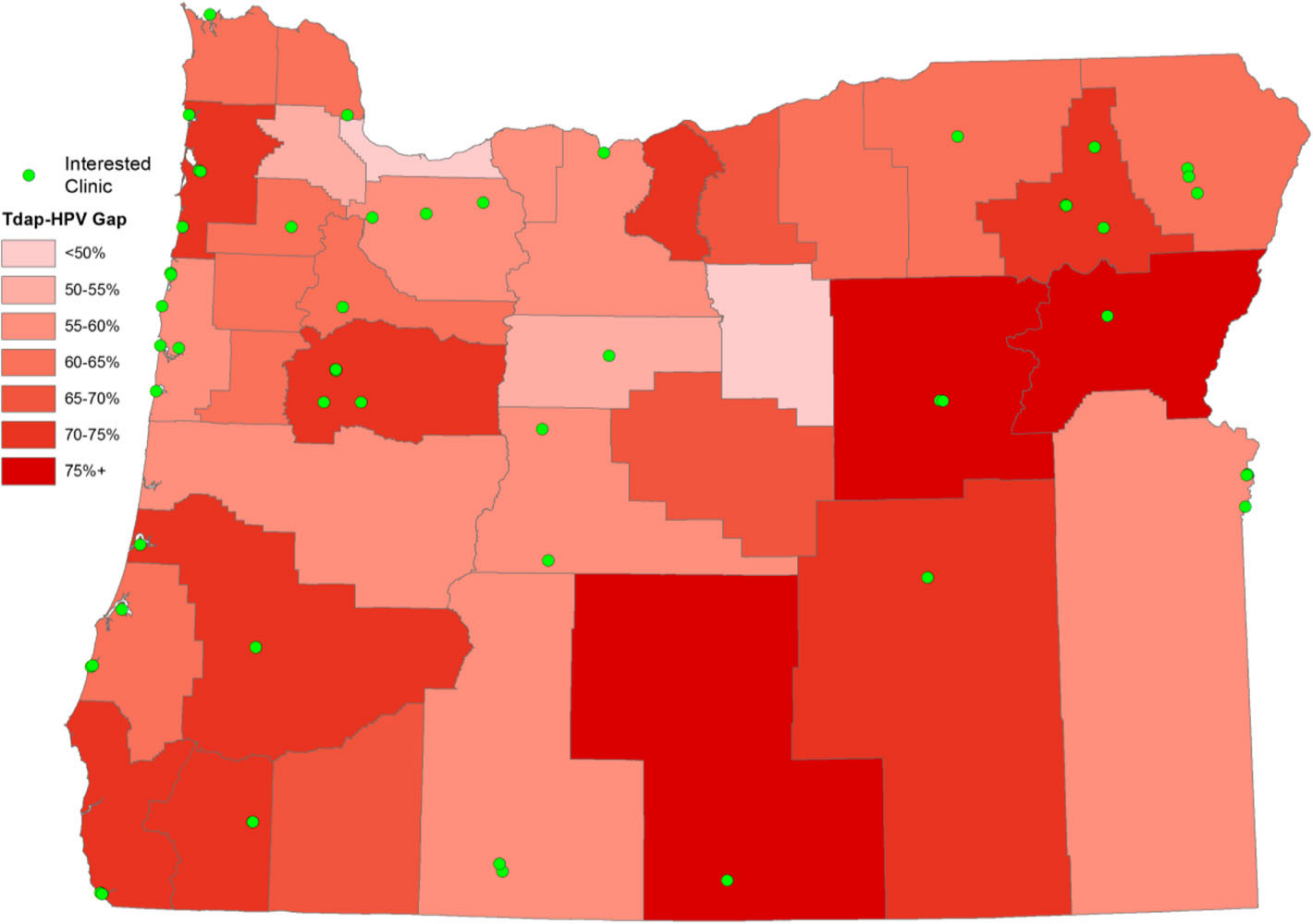
Differences in immunization rates between Tdap and HPV: minding the gap

Working with OIP, we have measured difference in immunization rates between Tdap (the only mandatory school vaccine, for adolescents grades 7–12, in Oregon), MenACWY (an optional school vaccine), and HPV. As shown in Fig. 3, many counties have a difference of greater than 50% for Tdap and MenACWY compared to HPV (Tdap is given but MenACWY and HPV vaccines are not). This demonstrates many missed opportunities for HPV vaccination.

## Discussion

Rural areas experience unique challenges regarding HPV vaccination and this phenomena is understudied in the current literature. Access to primary care can be challenging in rural areas and preventive care in these regions is often underutilized [30, 31]. Lower socioeconomic status, health literacy, and religiosity/conservatism are prevalent in rural areas [32] and may contribute to challenges with HPV vaccination. A study of communication disparities [33] found that rural parents are less likely than urban parents to engage in communication with their children’s healthcare clinicians, with lower rates of mutual information exchange, deliberation, and shared decision-making. A study, which used NIS-Teen data from 4124 parents of daughters aged 13–17 years, showed lower uptake of HPV vaccine in rural areas [10, 34]. Thus, studying these settings and implementing locally tailored interventions to enhance HPV vaccination uptake and series completion is critically important.

Our proposed study is innovative in at least nine important ways: (1) It is based in one of only two practice-based research network in the USA with a priority focus on rural primary care research; (2) it will involve tailored interventions for both pediatric and family medicine practices, allowing for specialty comparisons of staffing, workflow, and community linkages to deliver HPV vaccine; (3) ORPRN and OIP will collaborate to provide a descriptive baseline assessment of Oregon immunization rates and missed opportunities within rural practices, fostering a robust understanding of the gaps in meeting population health goals at practice and community levels (aim 1); (4) the practice-based intervention (aim 2) is designed to reduce burdens that busy primary care providers face using a team-based care approach with engaged, activated parents and adolescents; (5) the practice-based intervention (aim 2) is flexible enough to allow practices to tailor efforts that work best for them and they will not be burdened by data collection as the ALERT IIS system will provide vaccination data on all enrolled practices at 3-month intervals; (6) our interventions are designed to change primary care practice structure, communication strategies, and behavior through team-based care quality improvement (QI) efforts (see Fig. 1); (7) aim 3 focuses on how community-based organizations can synergistically improve the delivery of HPV vaccines in primary care practice, and will reveal how community-based primary care practices, local organizations, and state public health programs can collaborate to address a major population health concern; (8) the study design is very rigorous and evaluation methodologies use mixed-methods approaches; and (9) our findings will provide an important foundation for future dissemination research on tailoring interventions to patients, practices, and communities.

Important publications from this study are planned. They include (1) the use of the ALERT IIS as a data source for intervention studies, (2) comparing Medicaid and ALERT IIS data sources, (3) rates of HPV vaccinations of males and females in rural pediatric and family medicine practices, (4) urban/rural differences based on the practice location to see if these differences exist within practices, (5) urban/rural differences at the population and practice levels, (6) facilitators and barriers for both initiation and completion of HPV vaccines in rural pediatric and family medicine practices, (7) rates of missed opportunities to vaccinate for HPV according to youth gender, (8) effectiveness of a multi-component primary care-based intervention on completion of the HPV vaccine series and reduce missed opportunities to vaccinate, (9) characteristics of practices with high uptake and low uptake of the primary care-based quality improvement intervention, (10) contribution of evidence-based social marketing campaigns as a supplement to practice-based interventions to improve HPV vaccination, and (11) components and dissemination of a toolkit designed to help primary care practices improve HPV vaccination. In conclusion, there is a critical need to conduct this study, which started in July of 2018. We look forward to undertaking this important work and what we will learn from it.

## Abbreviations

ACOs: Accountable Care Organizations
CAC: Community Advisory Council
CCOs: Coordinated Care Organizations
CDC: Centers for Disease Control
GLMMs: Generalized linear mixed models
HPV: Human papillomavirus
IIS: Immunization Information System
MenACWY: Meningococcal ACWY
NIS: National Immunization Survey
OHA: Oregon Health Authority
OHSU: Oregon Health & Science University
OIP: Oregon Immunization Program
ORPRN: Oregon Rural Practice-based Research Network
QI: Quality improvement
QICA: Quality Improvement Change Questionnaire
Tdap: Tetanus, diphtheria, and acellular pertussis
